# Recurrent Great Saphenous Vein Reflux After Prior Endovenous Laser Therapy: Diagnostic and Management Challenges in Chronic Venous Insufficiency

**DOI:** 10.7759/cureus.116120

**Published:** 2026-09-11

**Authors:** Zuhayr Khan, Mahsum Jafri, Zohha Khan, Mehreen Khan, Constantino G Lambroussis

**Affiliations:** 1 General Medicine, Lake Erie College of Osteopathic Medicine, Elmira, USA; 2 Internal Medicine, Lake Erie College of Osteopathic Medicine, Elmira, USA; 3 Public Health, Saint Mary's University of Minnesota, Minneapolis, USA; 4 Osteopathic Medicine, Lake Erie College of Osteopathic Medicine, Elmira, USA

**Keywords:** chronic venous insufficiency (cvi), chronic venous leg ulcers, colored flow doppler ultrasound, endovenous laser therapy, great saphenous vein (gsv)

## Abstract

The comorbidity of chronic venous insufficiency is a prevalent vascular disorder that has further risks of progressing to ulceration and recurrent symptoms despite prior intervention. Endovenous laser therapy (EVLT) has become a first-line treatment for chronic venous insufficiency, especially in the axial great saphenous vein (GSV) reflux due to its high technical success and favorable safety profile. However, the recurrence of venous reflux remains clinically significant and often requires repeat imaging and intervention to further manage the condition. We present the case of a 54-year-old female patient with a history of chronic deep vein thromboses and prior EVLT of the right GSV who developed recurrent axial reflux associated with lateral malleolar ulceration. With duplex ultrasound mapping, we were able to demonstrate near-continuous reflux in the proximal GSV with incompetent accessory and perforator veins. Overall, this case report highlights the importance of comprehensive venous duplex imaging in identifying the mechanisms of recurrence and guiding management decisions, including the consideration of cyanoacrylate closure versus repeat thermal ablation.

## Introduction

Chronic venous insufficiency affects a substantial portion of the adult population and is characterized by venous hypertension, typically resulting from valvular incompetence and/or venous obstruction [1]. The clinical manifestations are broad and range from minor cosmetic varicosities to advanced diseases including edema, skin hyperpigmentation, lipodermatosclerosis, and ulceration [2]. Remarkably, the axial reflux of the great saphenous vein (GSV) represents the most common anatomic substrate of symptomatic chronic venous insufficiency [3].

Endovenous laser therapy (EVLT) has largely replaced traditional surgical stripping due to improved recovery time, reduced morbidity, and high occlusion rates [4,5]. This is shown in long-term studies, which demonstrate strong results in the majority of patients, with occlusion rates exceeding 90% at one year [6,7]. Despite these favorable outcomes, recurrence rates of 5% to 20% have been reported over longer follow-up periods [8]. The mechanisms of recurrence include recanalization of treated segments, neovascularization, untreated accessory veins, and incompetent perforators [9,10].

The use of duplex ultrasound remains the diagnostic cornerstone for both initial assessment and post-treatment surveillance. Accurate mapping is critical to identifying patterns of recurrence and directing subsequent therapy [11,12]. We present a radiology-focused case report of recurrent GSV reflux following prior EVLT, emphasizing the central role of duplex imaging in management planning.

## Case presentation

A 54-year-old female patient with a history of chronic deep vein thrombosis of the right lower extremity presented with progressive symptoms of venous insufficiency. She had previously undergone EVLT of the right GSV four years ago with initial symptomatic improvement. However, over the subsequent two years, she experienced increasing right lower extremity heaviness, which presented with evening-predominant swelling and the development of a large lateral malleolar ulcer. Throughout this sequence of events, she remained compliant with compression therapy.

During the physical examination, extensive tortuous varicosities extending across the anterior and lateral thigh and down to the calf were revealed. A large ulcer was present at the right lateral ankle with associated hemosiderin deposition and skin hyperpigmentation consistent with advanced chronic venous disease (Figure 1).

**Figure 1:**
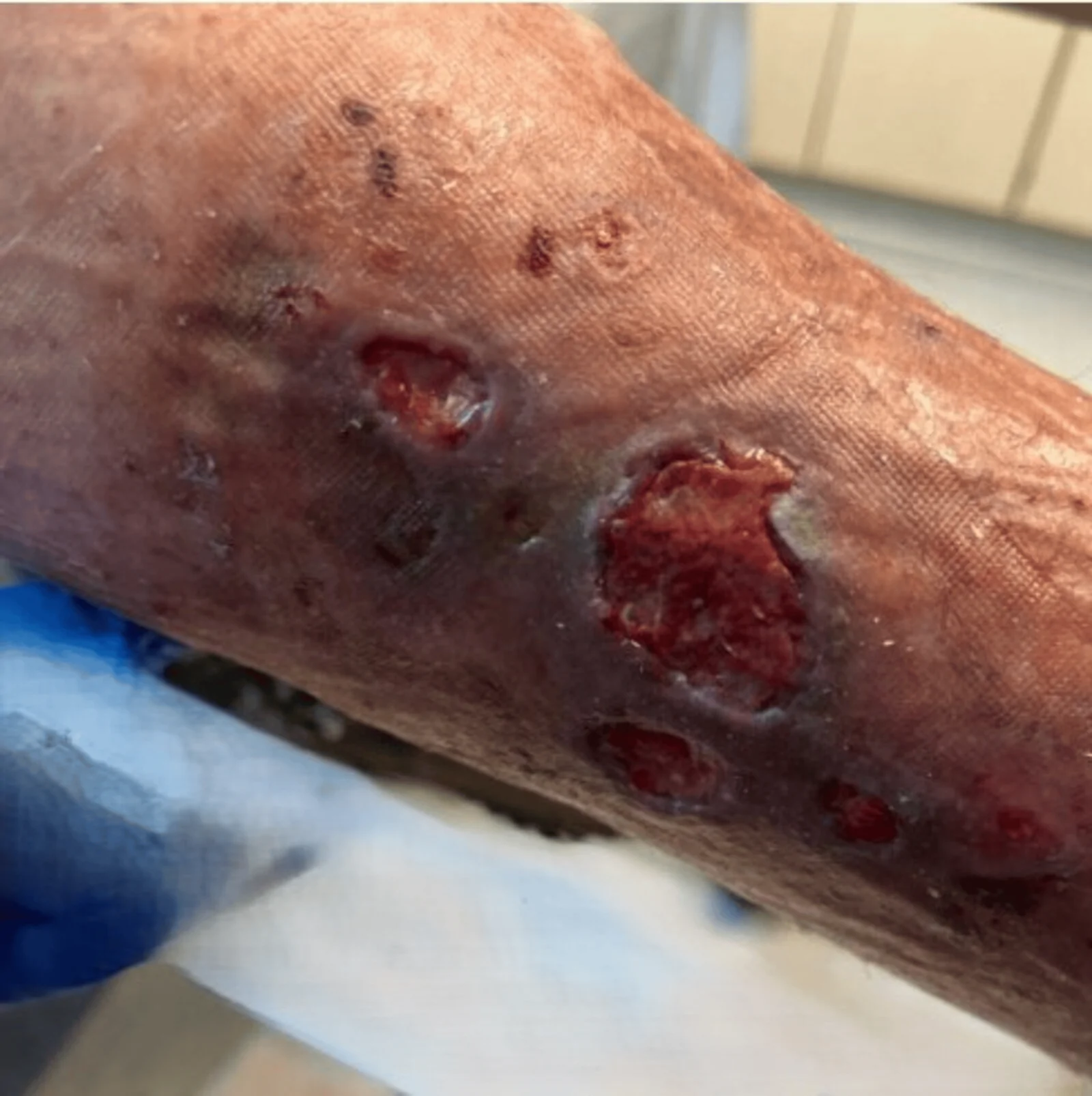
Large ulcer present at right lateral ankle due to chronic venous disease

There was 2+ pitting edema extending to the knee. Distal pulses were palpable and symmetric bilaterally. The contralateral extremity demonstrated smaller scattered varicosities without ulceration.

Given her prior intervention and worsening symptoms, a comprehensive venous duplex ultrasound with mapping was performed (Figure 2).

**Figure 2:**
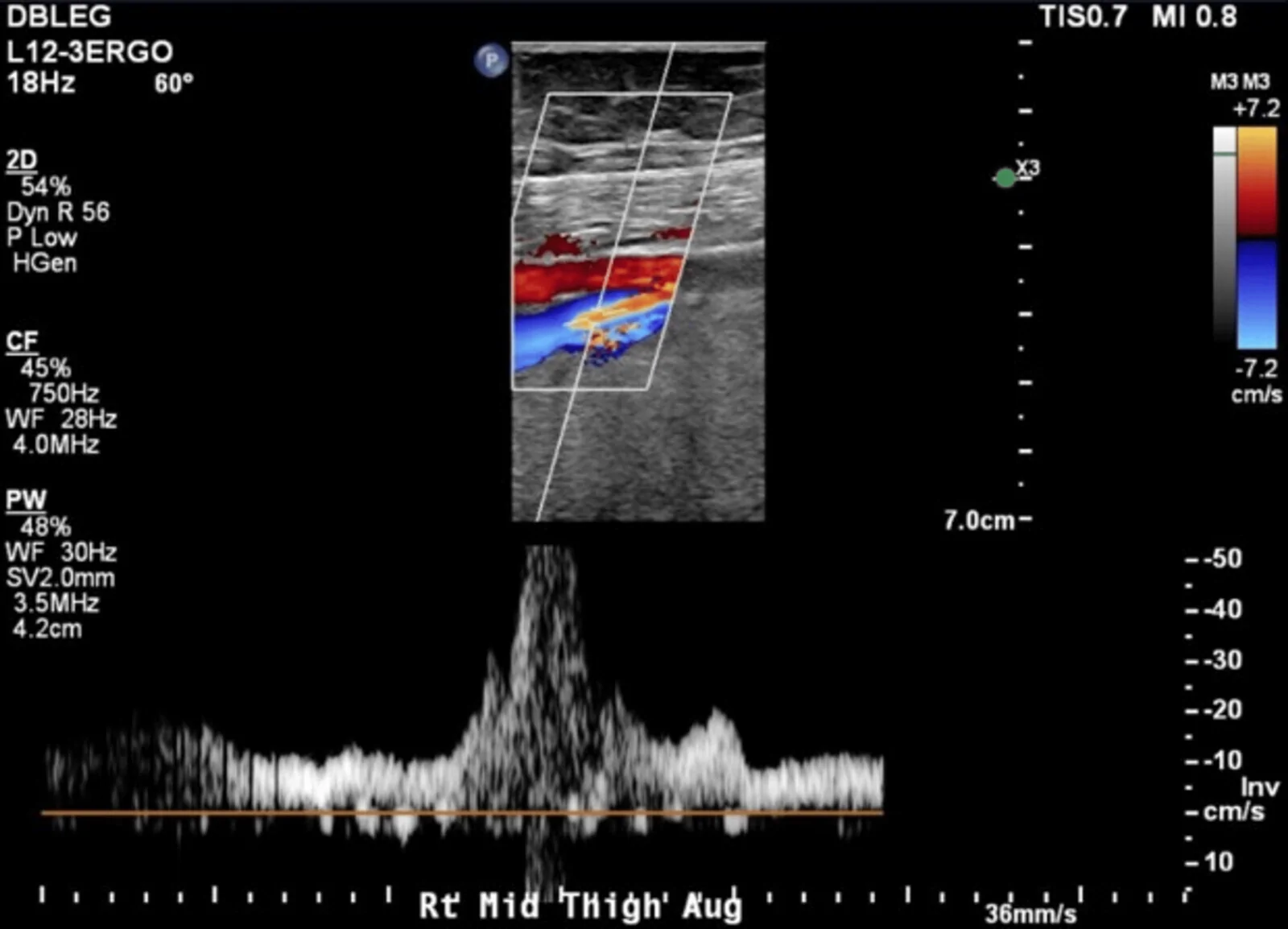
Right mid-thigh venous duplex ultrasound with spectral Doppler during distal augmentation Demonstrates antegrade flow followed by sustained retrograde flow below the baseline, consistent with pathologic venous reflux of the great saphenous vein and chronic venous insufficiency.

Evaluation included venous compressibility, spectral Doppler assessment, reflux testing with Valsalva maneuver and distal augmentation, vein diameter measurements, and assessment of reflux duration. The imaging findings using duplex doppler evaluation demonstrated a patent deep venous system with normal compressibility and no evidence of acute or chronic deep vein thrombosis. Bilateral arterial waveforms were normal and triphasic. Evaluation of the superficial venous system revealed near-continuous reflux of greater than three seconds in the proximal right GSV with the Valsalva maneuver. The GSV measured approximately 10 mm at its origin and tapered to 5 mm distally. The previously treated distal segment appeared closed. Significant reflux was identified within accessory veins of the lateral thigh and calf. The right small saphenous vein measured 3 mm and demonstrated no reflux. Although her prior deep vein thrombosis history may have contributed to chronic venous disease, the current duplex examination demonstrated a patent deep venous system without evidence of acute or chronic deep vein thrombosis. Overall, the findings were consistent with recurrent axial GSV reflux and chronic venous insufficiency of the right lower extremity.

Due to the presence of significant recurrent axial reflux within the right GSV and accessory tributaries, the patient was referred for repeat endovenous intervention. The proposed treatment plan includes targeting both the recanalized GSV and refluxing accessory veins. Treatment considerations included the prior EVLT, recurrent axial reflux, accessory vein involvement, ulceration, and the anatomy demonstrated on duplex imaging. A nonthermal option such as cyanoacrylate closure was considered because it avoids additional thermal exposure and tumescent anesthesia in a previously treated region. The anticipated outcome was improvement in venous hemodynamics, reduction in symptoms, and facilitation of ulcer healing. At the time of this report, the patient was still pending intervention.

## Discussion

Recurrent venous insufficiency following endovenous thermal ablation presents a diagnostic and therapeutic challenge. The process of recanalization of treated segments has been reported in a minority of cases and remains a recognized cause of recurrence [7,10]. In addition, untreated accessory anterior saphenous veins and incompetent perforators may serve as persistent sources of reflux, particularly in patients with advanced disease [3,9]. Recurrent disease may result from various etiopathogenesis such as recanalization, persistent/newly developed accessory vein reflux, incompetent perforating veins, and/or progression of underlying venous valvular dysfunction. Larger baseline GSV diameter has also been associated with an increased risk of treatment failure following endovenous laser ablation [13].

Duplex ultrasound is essential in differentiating these mechanisms. Pathologic reflux is defined as greater than 500 milliseconds of reversed flow in the superficial truncal veins (including the GSV), while greater than one second is considered diagnostic in the common femoral, femoral, and popliteal veins [11]. In this patient, greater than three seconds of GSV reflux substantially exceeded the diagnostic threshold for superficial venous incompetence. The comprehensive mapping of both axial and accessory pathways is essential in patients with prior intervention to avoid incomplete retreatment.

The management options for recurrent axial reflux include repeat thermal ablation or nonthermal alternatives such as cyanoacrylate closure. Repeating EVLT carries theoretical risks of nerve injury, skin burns, and fibrosis in previously treated segments [14]. The use of cyanoacrylate closure has demonstrated noninferiority to radiofrequency ablation and offers the advantage of avoiding tumescent anesthesia and thermal injury [15]. The long-term data supports durable occlusion rates with cyanoacrylate systems [15]. In this patient, a nonthermal approach may be particularly useful given the history of prior thermal ablation and recurrent reflux within a previously treated region.

In this case, imaging findings were central to management planning. An important limitation of this report is that the patient was still awaiting repeat intervention at the time of manuscript preparation. Therefore, treatment efficacy, symptom improvement, and ulcer healing outcomes are not yet available. Nonetheless, early identification and comprehensive imaging assessment may reduce symptom progression and improve patient outcomes.

## Conclusions

Recurrent GSV reflux following prior EVLT represents an important cause of persistent chronic venous insufficiency and ulcer recurrence. Detailed duplex ultrasound mapping is essential for identifying the mechanism of recurrence and guiding targeted intervention. Evaluation of both the treated GSV and associated accessory or perforating veins can help identify persistent sources of pathologic reflux. Radiologic evaluation therefore remains central to clinical decision-making in patients with complex recurrent venous disease. In selected patients, repeat thermal ablation or nonthermal approaches such as cyanoacrylate closure may be considered based on the anatomy and prior treatment history. Early identification and comprehensive imaging assessment may reduce symptom progression and improve patient outcomes.
